# Association between monocyte to high-density lipoprotein cholesterol ratio and bicuspid aortic valve degeneration

**DOI:** 10.3906/sag-2006-60

**Published:** 2020-08-26

**Authors:** Bilge DURAN KARADUMAN, Hüseyin AYHAN, Telat KELEŞ, Engin BOZKURT

**Affiliations:** 1 Department of Cardiology, Faculty of Medicine, Atılım University, Medicana International Ankara Hospital, Ankara Turkey; 2 Department of Cardiology, Faculty of Medicine, Ankara Yıldırım Beyazıt University, Ankara City Hospital, Ankara Turkey; 3 Department of Cardiology, Medicana International Ankara Hospital, Ankara Turkey

**Keywords:** Bicuspid aortic valve, tricuspid aortic valve, aortic stenosis, monocyte to high-density lipoprotein cholesterol ratio, inflammation

## Abstract

**Background/aim:**

From a pathophysiological point of view, inflammation is thought to be more dominant in bicuspid aortic valve (BAV) stenosis than tricuspid aortic valve (TAV) stenosis. Our study aimed to determine the association between monocyte to high-density lipoprotein cholesterol (HDL-C) ratio (MHR), a new inflammatory marker, and the speed of progression of stenosis and pathophysiology of BAV stenosis.

**Materials and methods:**

A total of 210 severe aortic stenosis patients (70 consecutive BAV patients, 140 matched TAV patients) were retrospectively enrolled in the study. Clinical and echocardiographic data and laboratory results related to our research were collected retrospectively from the patients’ records. MHR was measured as the ratio of the absolute monocyte count to the HDL-C value.

**Results:**

Seventy BAV (mean age: 72.0 ± 9.1 years, 42.9% female) and 140 TAV patients (mean age: 77.9 ± 8.3 years, 51.4% female) with severe aortic stenosis were enrolled in this study. There was no difference between the two groups in terms of another baseline demographic or clinic findings except age (P < 0.001). Monocyte count, hemoglobin level, mean platelet volume was significantly higher, and HDL-C level was significantly lower in the BAV group, while other lipid and CBC parameters were found to be similar. In the multivariate analysis, MHR (P = 0.005, 95% CI: 0.90–0.98) and, as expected, age (P = 0.001, 95% CI: 1.02–1.11) were found to be significant as the independent predictor of BAV, after adjusting for other risk factors.

**Conclusion:**

Our study showed a significant correlation between increased MHR and BAV. MHR was determined as a significant independent predictor for the speed of progression and diagnosis of severe BAV stenosis in multivariate analysis.

## 1. Introduction

Bicuspid aortic valve (BAV) is the most common congenital heart anomaly in adults, and its prevalence has been reported to be 1–2% [1]. BAV usually coexists with other obstructive diseases such as aortic coarctation, or with hypoplastic left heart, interrupted aortic arch, and noncompaction cardiomyopathy [2]. BAV is associated with a faster aortic valve degeneration, resulting in aortic valve stenosis (AS), or insufficiency [3].

Calcific aortic valve disease is progressive and present in 20–30% of patients aged over 65 years, and 48% of patients over 85 years, also AS affects 2–3% of patients under 65 years and 8% of patients over 85 years [4]. Calcific AS was thought to be a passive and degenerative process that progresses with age. Still, recent data indicate that chronic inflammation plays a role, similar to the pathophysiology of coronary atherosclerosis [5]. Albeit a genetic predisposition has been detected in the development of tricuspid aortic valve (TAV) stenosis, besides the mechanical stress caused by minor changes in cusp size; the combination of atherosclerotic risk factors such as diabetes mellitus (DM), hypertension (HT), hyperlipidemia (HL), age, and smoking habit plays a role in the onset of valve stenosis [6,7]. 

Compared to TAV stenosis, BAV stenosis develops earlier in life. Over recent years, significant attention has been paid to explain BAV pathogenesis and complications, including the addition of genetics and inflammation. Atherosclerotic risk factors such as hyperlipidemia (HL) and HT are associated with the development of AS in BAV [8]. Moreover, some but not all researchers have presented evidence of systemic inflammatory activation in BAV [9].

Monocyte activation plays a vital role in chronic inflammation and cardiovascular diseases by modulating inflammatory cytokines [10]. High-density lipoprotein cholesterol (HDL-C) is highly valuable in inhibiting endothelial expression of adhesion molecules and preventing monocyte uptake into the arterial wall. Therefore, the monocyte-to-HDL-C ratio (MHR) is a simple estimation method to predict inflammation and reported as a significant predictor of cardiovascular diseases in recent studies [11]. Thus, this study is aimed to analyze the potential role of typical inflammatory markers like MHR on the diagnosis, the speed of progression, diagnosis of severe BAV stenosis, and pathophysiology of BAV stenosis.

## 2. Materials and methods

### 2.1. Study population

We enrolled retrospectively 70 consecutive BAV patients who were admitted to our clinic between January 2012 and October 2018 along with 140 sex, DM, HT, HL, body mass index matched TAV patients diagnosed with severe AS. According to echocardiographic findings, we thought that BAVs were isolated congenital lesions. Baseline characteristics of patients, clinical and echocardiographic data, and laboratory results related to our study were collected retrospectively from the hospital information management system as well as patient files. When echocardiographic results were examined; those who met these criteria—aortic valve area (AVA) <1.0 cm2 or mean gradient >40 mmHg or maximum jet velocity >4.0 m/s, AVA index <0.6 cm2/m2—were considered as severe AS. Diagnosis of the BAV was made by visualizing two aortic leaflets clearly in the transthoracic parasternal short-axis image, irrespective of the presence of raphe. The BAV and TAV patients with presence of rheumatic or congenital disease, severe mitral/tricuspid valve disease, acute or history infective endocarditis, obstructive coronary artery disease, hemodynamically significant arrhythmias, malignancies, hepatic, renal or hematologic severe diseases including leucocythemia and acute or chronic infection or inflammatory condition were excluded from the study. The local ethics committee approved the study.

### 2.2. Laboratory examination of the blood

Peripheral blood samples were drawn from a large antecubital vein for detection of complete blood count (CBC), total serum cholesterol (TC), triglycerides (TGs), HDL cholesterol, and low-density lipoprotein (LDL) cholesterol and plasma glucose (Symex K-1000, Kobe, Japan) on admission. All routine biochemical analyses were performed using an autoanalyzer (Roche Diagnostic Modular Systems, Tokyo, Japan). Monocyte count was defined by differential analysis of complete blood count, and the MHR was measured as the ratio of the absolute monocyte count to the HDL-cholesterol value. NLR was calculated as the ratio of the absolute neutrophil count to the lymphocyte count. In our laboratory, the reference value for monocyte was 0–0.9 K/uL.

### 2.3. Statistical analysis

All statistical analyses were applied in SPSS version 22.0 Windows (SPSS Inc, Chicago, Illinois). A two-tailed P-value <0.05 was considered statistically significant. Categorical variables are presented as numbers and percentages and were compared by use of the chi-square test and Fisher’s exact test. Continuous variables are expressed as mean ± SD and were compared normally distributed variables with Student’s t-test and nonnormally distributed variables with the Wilcoxon rank-sum test. Multivariable logistic regression analyses were performed for all the variables that were statistically different in the univariate analysis to identify the independent risk factors of diagnosis BAV in the study population.

## 3. Results

Seventy BAV (mean age: 72.0 ± 9.1 years, 42.9% female) and 140 TAV patients (mean age: 77.9 ± 8.3 years, 51.4% female) with severe AS were enrolled in this study. The patients in the BAV group were younger than those in the TAV group (P < 0.001); besides this, there was no difference between the two groups in terms of another baseline demographic or clinic findings. Comparison of baseline demographic, clinical, and laboratory data between BAV and TAV patients were represented in Table 1.

**Table 1:** Baseline clinical and laboratory parameters.

Parameters	All patients n = 210	Group 1 BAV n = 70	Group 2 TAV n = 140	P-value
Age (years)	75.9 ± 9.0	72.0 ± 9.1	77.9 ± 8.3	<0.001
Female n (%)	102 (48.6)	30 (42.9)	72 (51.4)	0.241
BMI (kg/m2)	27.1 ± 4.7	27.2 ± 4.8	27.0 ± 4.6	0.801
NYHA n (%) - 2 - 3 - 4	64 (30.5) 123 (58.5) 23 (11.0)	20 (28.6) 42 (60.0) 8 (11.4)	44 (31.4) 81 (65.9) 15 (10.7)	0.711
DM n (%)	66 (31.4)	27 (38.6)	39 (27.9)	0.115
HT n (%)	171 (81.4)	53 (75.7)	118 (84.3)	0.132
HL n (%)	112 (53.3)	35 (50.0)	77 (55.0)	0.494
Current smoker n (%)	58 (27.6)	18 (25.7)	40 (28.5)	0.684
Previous PCI n (%)	45 (21.4)	16 (22.9)	29 (20.7)	0.721
Previous CABG n (%)	55 (26.2)	19 (27.1)	36 (25.7)	0.824
Previous MI n (%)	27 (12.9)	12 (17.1)	15 (10.7)	0.190
Moderate to severe COPD n (%)	87 (41.4)	44 (48.6)	53 (37.9)	0.315
AF n (%)	46 (21.9)	14 (20.0)	32 (22.9)	0.637
Stroke n (%)	14 (6.7)	3 (4.3)	11 (7.9)	0.328
CAD - Normal - Nonobstructive	60 (28.6) 90 (42.9)	21 (30.0) 32 (45.7)	39 (27.9) 58 (41.4)	0.057
Medication n (%) - RAS blocker - Statin - Beta blocker - ASA - OAC	153 (72.8) 106 (50.4) 80 (38.1) 151 (72.9) 49 (23.7)	45 (64.2) 28 (40.0) 23 (32.8) 54 (79.4) 12 (17.6)	108 (77.1) 78 (55.7) 57 (40.7) 97 (69.8) 37 (26.7)	0.135

BMI: Body Mass Index; NYHA: New York Heart Association; DM: Diabetes Mellitus; HT: Hypertension; PCI: Percutaneous Coronary Intervention, CABG: Coronary artery Bypass Grafting, MI: Myocardial Infarction, COPD: Chronic Obstructive Pulmonary Disease, AF: Atrial Fibrillation; CAD: Coronary Artery Disease; RAS: Renin-angiotensin-system; ASA: Acetyl salicylic acid; OAC: Oral anticoagulant

Monocyte count, hemoglobin level, mean platelet volume were significantly higher, and HDL-C level was significantly lower in the BAV group, while other lipid and CBC parameters were found to be similar. Nevertheless, a numerically higher rate of NLR was found in the BAV group compared with the TAV group, and MHR was statistically significantly higher in the BAV group (17.6 ± 10.1 vs. 12.4 ± 6.6, P = <0.001). Laboratory parameters are shown in Table 2.

**Table 2:** Laboratory parameters.

Parameters	All patients n = 210	Group 1 BAV n = 70	Group 2 TAV n = 140	P-value
Serum glucose mg/dL	126.1 ± 52.8	135.2 ± 69.3	121.6 ± 41.8	0.079
HbA1c (%)	6.23 ± 1.2	6.54 ± 1.5	6.15 ± 0.9	0.084
Creatinine (mg/dL)	1.01 ± 0.40	0.97 ± 0.30	1.03 ± 0.44	0.301
Total cholesterol (mg/dL)	167.4 ± 43.6	159.8 ± 43.0	171.2 ± 43.7	0.074
Triglyceride (mg/dL)	122.8 ± 69.8	118.7 ± 59.0	124.9 ± 74.8	0.543
LDL cholesterol (mg/dL)	98.6 ± 35.3	94.7 ± 39.4	100.6 ± 33.0	0.249
HDL cholesterol (mg/dL)	45.4 ± 13.2	42.1 ± 13.7	47.0 ± 12.7	0.012
Hemoglobin (mg/dL)	11.7 ± 2.0	12.1 ± 1.7	11.4 ± 2.1	0.023
Neutrophil count, (× 10^3^/L)	4.80 ± 1.78	5.08 ± 1.82	4.65 ± 1.75	0.098
Lymphocyte count (× 10^3^/L)	1.59 ± 0.62	1.68 ± 0.61	1.55 ± 0.62	0.147
NLR	3.41 ± 1.77	3.54 ± 2.09	3.34 ± 1.59	0.441
Monocyte count (× 10^3^/L)	0.59 ± 0.27	0.65 ± 0.26	0.55 ± 0.27	0.010
MHR	44.6 ± 14.2	17.6 ± 10.1	12.4 ± 6.6	<0.001
Platelet count (× 10^3^/L)	237.9 ± 81.3	243.4 ± 96.8	235.1 ± 72.6	0.488
MPV (fL)	10.5 ± 1.2	10.8 ± 1.2	10.4 ± 1.2	0.034
RDW	15.3 ± 2.1	15.2 ± 1.7	15.4 ± 2.2	0.598

NLR: neutrophil to lymphocyte ratio; MHR: monocyte to HDL-C ratio; RDW: red cell distribution width

Table 3 presents comparison of baseline echocardiographic parameters of all groups. There was no significant difference observed between the groups concerning left ventricle ejection fraction (LVEF), aortic mean gradient, and aortic valve area (P > 0.05); however, left ventricular end-diastolic diameter, left ventricular end-systolic diameter, aortic annulus, and systolic pulmonary artery pressure (sPAP) were different between the groups.

**Table 3:** Comparison of baseline echocardiographic parameters.

Parameters	All patients n = 210	Group 1 BAV n = 70	Group 2 TAV n = 140	P-value
LVEF (%)	49.8 ± 14.8	47.3 ± 16.4	51.0 ± 13.8	0.082
LVEDD (cm)	4.83 ± 0.72	4.97 ± 0.73	4.75 ± 0.71	0.041
LVESD (cm)	3.26 ± 0.92	3.45 ± 1.03	3.17 ± 0.85	0.039
Septal wall thickness (cm)	1.39 ± 0.24	1.39 ± 0.27	1.39 ± 0.22	0.814
Posterior wall thickness (cm)	1.30 ± 0.19	1.29 ± 0.23	1.30 ± 0.16	0.684
Aortic annulus (cm)	2.19 ± 0.22	2.26 ± 0.24	2.15 ± 0.21	0.001
LA (cm)	4.72 ± 0.69	4.62 ± 0.60	4.77 ± 0.73	0.171
Aortic peak velocity (m/s)	4.4 ± 0.5	4.4 ± 0.6	4.4 ± 0.5	0.711
Aortic max gradient (mmHg)	80.5 ± 20.7	81.0 ± 21.6	80.3 ± 20.3	0.815
Aortic mean gradient (mmHg)	49.4 ± 13.4	50.4 ± 14.0	48.9 ± 13.0	0.442
AVA (cm^2^)	0.67 ± 0.16	0.69 ± 0.18	0.66 ± 0.15	0.235
AVA index (cm^2^)	0.37 ± 0.09	0.38 ± 0.10	0.36 ± 0.08	0.416
sPAP (mmHg)	43.6 ± 17.4	39.9 ± 16.5	45.5 ± 17.6	0.028

LVEF: Left Ventricular Ejection Fraction, LVEDD: Left Ventricular End Diastolic Diameter, LVESD: Left Ventricular End Systolic Diameter, LA: Left Atrium, LVH: Left Ventricular Hypertrophy, AVA: Aortic Valve Area, sPAP: systolic Pulmonary Artery Pressure

In the multivariate analysis, MHR (P = 0.005, 95% CI: 0.90–0.98) and, as expected, age (P = 0.001, 95%CI: 1.02–1.11) were found to be significant as the independent predictor of BAV, after adjusting for other risk factors (Table 4). Moreover, NLR, LVEF, and other lipid parameters had no predictive value for BAV compared to TAV stenosis.

**Table 4:** Multivariate logistic regression analysis to detect independent variables for the diagnosis of the bicuspid aortic valve.

Parameters	Odds ratio	95% CI	P-value
Sex	0.87	0.41–1.81	0.716
Age	1.07	1.02–1.11	0.001
Total cholesterol	1.02	0.99–1.04	0.085
LDL cholesterol	0.98	0.95–1.00	0.206
NLR	1.02	0.85–1.24	0.780
MHR	0.94	0.90–0.98	0.005
DM	1.04	0.38–2.83	0.939
HT	1.82	0.66–4.98	0.241
Glucose	0.99	0.98–1.00	0.471
LVEF	1.00	0.98–1.03	0.649

NLR: neutrophil to lymphocyte ratio; MHR: monocyte to HDL-C ratio; DM: Diabetes Mellitus; HT: Hypertension; LVEF: Left Ventricular Ejection Fraction

## 4. Discussion

The main findings of our study are: 1) The MHR was found to be significantly higher in patients with BAV compared to TAV; 2) BAV patients were younger than TAV patients; and 3) MHR was determined as a significant independent predictor for diagnosis of severe BAV stenosis in multivariate analysis.

The underlying eccentric valve structure and leaflet matrix in the bicuspid aortic valve expose the cusps to high mechanical and functional stress and inevitably cause aortic stenosis [12,13]. Although pathology in the bicuspid aorta begins with a congenital anomaly, the progression of the disease continues with calcification, atherosclerosis, and the inflammatory process, as in TAV [14]. However, it is not effortless to understand which of these processes has a large share or in what order it affects. Inflammation may have the most crucial role in BAV degeneration. Inflammatory process maintained by the presence of increased shear stress includes various mechanisms such as the collection of inflammatory cells and release of cytokines, oxidative stress, and endothelial dysfunction [15]. Various mechanisms, in different forms of the BAV, may be heterogeneously responsible. While oxidative stress is in the foreground in Type 1 BAV, endothelial dysfunction becomes vital in Type 2 BAV [16]. However, they all merge in the inflammatory process, which can cause subsequent changes, such as tissue remodeling and calcification. 

Few studies have compared clinical and pathopsychological profiles in age-matched cohorts between BAV and TAV stenosis. Differences between BAV and TAV stenosis, such as the predominance of associated cardiovascular risk factors, associated aortapathyes, or variations in the size of the aortic annulus, may affect clinical outcomes of aortic valve replacement [17]. Additionally, it should be noted that the reason for the occurrence of AS at an earlier age in BAV may be that the inflammatory process may be more dominant than TAV. In our study, although traditional risk factors are similar except for the patients’ ages, the earlier development of degeneration in the BAV group also supports the role of inflammation.

Monocytes are the reservoir of several cytokines and molecules that associate with endothelial cells, attending to aggravation of inflammatory pathways [18]. Inflammation plays an essential role in the development and progression of atherosclerosis, and its effect in many disease pathophysiologies has been demonstrated. Since tissue macrophages originate from circulating monocytes, the number of circulating monocytes and monocyte subsets with different properties has attracted attention in both atherosclerosis and valvular disease studies [19]. Moreover, HDL reduces the prooxidant and proinflammatory consequences of monocytes by preventing the displacement of macrophages and oxidation of low-density lipoprotein molecules, and also by promoting the flow of cholesterol from these cells [20]. For the reasons mentioned above, it is very reasonable to use a single marker, MHR, as an inflammatory marker by proportioning these two separate markers, monocyte, and HDL-C.

MHR was found to be related to cardiovascular diseases [21], including stent thrombosis in STEMI [22], coronary artery ectasia [23], and bare-metal stent restenosis [24]. A significant association between MHR levels and inflammation was demonstrated by the study of Acikgoz et al. [25]; thus, it has been extensively investigated in other diseases associated with inflammation. Moreover, it has been shown to be a new potential marker for predicting abdominal aortic aneurysm (AAA) size in asymptomatic patients. Çağlı et al. showed that MHR was independently associated with the AAA size after dividing 120 asymptomatic AAA patients into two groups according to the MHR median value (54.3 ± 10.6 mm vs 62.0 ± 12.4 mm, P < 0.001, respectively) [26].

Diagnostic value of MHR has been reported by Demir et al. in rheumatic mitral stenosis (RMS) [27]. In their study, as mentioned earlier, in which 368 patients with RMS were included, it was shown that MHR increased in proportion to the severity of stenosis in the RMD group compared to the control group. They found MHR (odds ratio: 1.118 [95% CI: 1.018–1.228]; P = 0.019) as the independent predictor of the presence of RMS together with the left atrial diameter and CRP. Similarly, Pamukcu et al. demonstrated that the presence of mitral annular calcification was associated with higher MHR, and MHR was significantly correlated with mitral annular calcification [28].

The purpose of our study was to describe the relationship between MHR, which effectively predicts inflammation and severe BAV stenosis. Compatible with previous studies on mitral stenosis, increased MHR was found to be related to the BAV, in which inflammation plays an essential role in its pathophysiology. However, in our study, unlike Demir et al.’s [27], rheumatic severe aortic stenosis was not included in the study. Bicuspid and tricuspid AS patients were compared, but inflammation plays a role in the pathophysiology of both these two groups. We hypothesized that inflammation in BAV stenosis is more severe than TAV stenosis. Nevertheless, in similarity with our results, inflammation may be considered to be more prevalent in BAV, because most of the time, BAV is not alone, it should be regarded as part of the ‘bicuspid aortic valve syndrome’. In the present study, no significant difference was found between the two groups in terms of another important marker of inflammation, NLR. In most NLR studies, disease and control groups were compared and higher in NLR disease groups. In our study, both groups consisted of severe aortic stenosis groups where inflammation played an important role. In addition, another explanation may be that the primary purpose of the present study was not to compare NLR. Another inflammation-related parameter, MPV, was statistically higher in the BAV group. Elevated MPV means more substantial platelet size, a pointer of platelet activations and functions that are considered a risen risk of cardiovascular disorder. Prior researches have revealed that platelet activation increased since shear stress caused by turbulence flow in the stenotic valve in AS patients [29,30].

In our study, as in other studies, severe AS developed in the BAV group at an earlier age than TAV. Similar to our study, echocardiographic studies have shown that degeneration typically begins in the second decade in most middle-aged patients with BAV stenosis [31]. This early degeneration may be linked to more aggressive inflammatory changes secondary to augmented macrophage infiltration and neovascularization [32].

Our study has some limitations. Firstly, it was a single-center retrospective study. Secondly, we measured MHR only at baseline rather than the use of several measurements taken at different time points, so serial MHR variations were not evaluated. Thirdly, the impact of other inflammatory markers, such as CRP, has not been evaluated due to a lack of data. Finally, due to the retrospective design of the study, we did not have information about diabetes drugs that may affect inflammation parameters.

In conclusion, to our knowledge, our study showed the important relationship between monocyte to HDL-C ratio and bicuspid aortic valve for the first time in the literature. MHR can be calculated easily from the routine blood parameters, and also this parameter can be an essential predictor for patients with BAV who have a high inflammatory risk and a high rate of progression to severe aortic stenosis. In light of our findings, large-scale prospective studies are needed to investigate the effect of antiinflammatory treatments on early BAV stenosis.
